# Extracellular Polymeric Substances Produced by the Thermophilic Cyanobacterium *Gloeocapsa gelatinosa*: Characterization and Assessment of Their Antioxidant and Metal-Chelating Activities

**DOI:** 10.3390/md20040227

**Published:** 2022-03-26

**Authors:** Wejdene Gongi, Juan Luis Gomez Pinchetti, Nereida Cordeiro, Hatem Ben Ouada

**Affiliations:** 1 Laboratory of Blue Biotechnology & Aquatic Bioproducts, National Institute of Marine Sciences and Technologies, Monastir 5000, Tunisia; hatbenouada@gmail.com; 2 French Guyana University, UMR 228 Espace-Dev, 97300 Cayenne, France; 3 Banco Español de Algas (BEA), Instituto de Oceanografía y Cambio Global, Universidad de Las Palmas de Gran Canaria, Muelle de Taliarte s/n, 35214 Telde, Spain; juan.gomez@ulpgc.es; 4 LB3-Faculty of Science and Engineering, University of Madeira, 9020-105 Funchal, Portugal; ncordeiro@staff.uma.pt; 5 CIIMAR—Interdisciplinary Centre of Marine and Environmental Research, University of Porto, 4450-208 Matosinhos, Portugal

**Keywords:** *Gloeocapsa gelatinosa*, extracellular polymeric substances, physicochemical characterization, functional properties, antioxidant activity, extremophilic, cyanobacteria

## Abstract

Cyanobacteria, particularly thermophilic strains, represent an important potential source of EPSs, harboring structural complexity that predicts diverse and specific bioactive potential. The thermophilic cyanobacteria *Gloeocapsa gelatinosa,* isolated from a natural hot source in Ain Echfa (Tunisia), was cultivated in a cylindrical reactor, and the production of biomass and EPSs was investigated. Results revealed that the strain is amongst the most efficient EPSs producers (0.89 g L^−1^) and that EPSs production was not correlated with the growth phase. EPSs were sulfated heteropolysaccharides containing carbohydrates (70%) based on nine different monosaccharides, mainly mannose (22%), and with the presence of two uronic acids. EPSs were formed by two polymers moieties with a molecular weight of 598.3 ± 7.2 and 67.2 ± 4.4 kDa. They are thermostable in temperatures exceeding 100 °C and have an anionic nature (zeta potential of −40 ± 2 mV). Atomic force microscopy showed that EPSs formed multimodal lumps with 88 nm maximum height. EPSs presented high water holding capacity (70.29 ± 2.36%) and solubility index (97.43 ± 1.24%), and a strong bivalent metal sorption capacity especially for Cu^2+^ (91.20 ± 1.25%) and Fe^2+^ (75.51 ± 0.71%). The antioxidant activity of *G. gelatinosa* EPSs was investigated using four methods: the β-carotene-bleaching activity, DPPH assays, iron-reducing activity, and metal-chelating activity. EPS has shown high potential as free radicals’ scavenger, with an IC_50_ on DPPH (0.2 g L^−1^) three-fold lower than ascorbic acid (0.6 g L ^−1^) and as a metal chelating activity (IC_50_ = 0.4 g L^−1^) significantly lower than EDTA. The obtained results allow further exploration of the thermophilic *G. gelatinosa* for several biotechnological and industrial applications.

## 1. Introduction

The enlarged requirement for natural polymers or biopolymers for different industrial and biotechnological applications has led to a renewed interest in extracellular polymeric substances (EPSs) production by microalgae and mainly by cyanobacteria [1].

EPSs are a complex mixture of macromolecular polyelectrolytes, mainly consisting of polysaccharides, amine-group, halide-group, aliphatic alkyl group, aromatic compounds, and nucleic acids [2,3]. They have been identified as a new rich source of bioactive compounds, such as antiviral and anti-inflammatory, antitumor, anticancer [1], and wound healing [4] agents. It has also been shown that cyanobacterial EPSs have a distinct antioxidant activity [1,5] and metal chelating properties [6].

Cyanobacterial EPSs have been applied in foods, drug delivery, sludge settling and dewatering, and metal removal in mining and industrial waste treatments, among others [7]. They have been recognized as a potential alternative to conventional chemical polymers because of their biodegradability, biocompatibility, and non-toxicity [8]. In addition, cyanobacterial EPSs are easy to produce, and their simplicity and environmentally friendly recovery from liquid cultures make them one of the most attractive sources of new biopolymers [9].

Nevertheless, the production of EPSs is strain-dependent. The large-scale production of EPSs remains limited, and the search for new strains with strong growth rates in addition to the high productivity of EPSs with confirmed biological activities remains current and necessary.

During this last decade, a growing interest has been carried to the microorganisms thriving in the waters of the hydrothermal hot springs. In general, hot spring natural ecosystems, unfavorable for common organisms, are inhabited by specific photosynthetic microalgae and cyanobacteria, forming thick mats anchored in natural or artificial submerged boulders [10]. Although some eukaryotic species such as diatoms [11] and green algae, Chlorophyta [12] are reported in these harsh environments, prokaryotic ones, cyanobacteria, are the most prominent [13]. These species can be extreme thermophiles, which naturally grow at temperatures of 55–80 °C or, for the most, moderate thermophiles, preferably pushing below 40 °C but having the ability to resist temperatures above 60 °C [13].

According to many authors, organisms with high optimal growth temperatures have higher biomass productivity [4,12,14]. Furthermore, because of thriving at high temperatures, thermophiles cyanobacteria can possess vast possibilities for physiological adaptation and genetic modifications that make them potential producers of high-value thermo-stable bioactive compounds with higher production yields than mesophilic ones [12,15]. Hot temperature conditions induce the high production of EPSs involved in protecting the cells, the formation of biofilms, and/or the sequestration/immobilization of metal ions [8]. This provides a valuable resource for the isolation of new strains with great potential to produce EPSs and further present high versatility of applications as bioactive compounds.

The present study investigates the production of EPSs in laboratory cultures of a thermophilic cyanobacterium strain *Gloeocapsa gelatinosa* (*G. gelatinosa*) isolated from a Tunisian hot spring at 60 °C of temperature. Few data are available in the literature regarding the cyanobacteria *Gloeocapsa* EPSs. Patterson et al. [16] underline the potential of this genus as a source of antiviral compounds. It was also shown that the *Gloeocapsa* sp. exopolysaccharides inhibit the growth of bacteria and fungi strains [17]. However, to our knowledge, no studies intend the antioxidant potential of the EPSs produced by *G. gelatinosa*. The EPSs obtained in this work were characterized regarding their physicochemical characteristics (biochemical, elementary, and monosaccharide’s composition, proton nuclear magnetic resonance, infrared spectroscopy, molecular weight, zeta potential, and atomic force microscopy) and their functional properties (thermal stability, viscosity, water holding, and metal sorption capacities). Further antioxidant activity was assessed by measuring the total antioxidant activity, α,α diphenyl-β-picrylhydrazyl (DPPH) radical scavenging, iron-reducing, and metal-chelating activity.

## 2. Results and discussion

### 2.1. G. gelatinosa Biomass and EPSs Production

**Figure 1:**
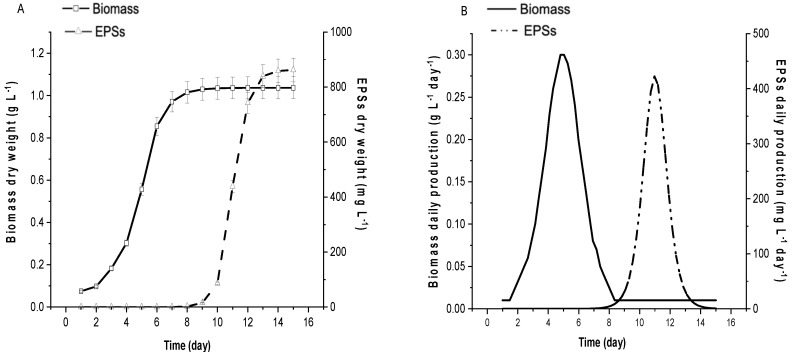
Biomass (full line) and EPSs (dot line) kinetics to *G. gelatinosa* at optimal growth condition: (**A**) production (g L^−1^) and (**B**) productivity (g L^−1^ day^−1^). Error bars represent standard deviation (*n* = 3).

The daily *G. gelatinosa* biomass production and EPSs kinetics (Figure 1A,B) exhibit a typical cyanobacterial growth curve with a clear exponential phase, observed from day 3 to day 7 of culture time with a maximum biomass density of 1.03 g L^−1^ and maximum daily productivity of 0.27 g L^−1^ day^−1^. The specific growth rate (µ) evaluated as 0.56 day^−1^ was significantly higher than that obtained, in optimized laboratory culture conditions, for *Arthrospira platensis*, considered as the main cultivated cyanobacteria at an industrial level due to its fast growth rate (µ =0.46 day^−1^ [18]). Concerning the *G. gelatinosa* EPSs, the maximum concentration (0.89 g L^−1^) was observed after 12 days of culture. The EPSs’ daily productivity reached 0.43 g L^−1^ day^−1^, which is within the range of the highest EPSs producers [1]. Indeed, the highest EPSs production might be related to the extreme environment (hot water) from where *G. gelatinosa* was isolated, which would lead this strain to possess efficient mechanisms for producing EPSs to preserve cells from desiccation and therefore from death [1]. Thus, the obtained results emphasize the ability of the cyanobacterium *G. gelatinosa* to combine high biomass and EPSs production.

Moreover, as shown in Figure 1B, to *G. gelatinosa,* a very limited EPSs production was detected at the exponential biomass growth phase (<1% of dry biomass weight). However, as the culture approached the stationary phase, the EPSs content increased. The maximum daily biomass productivity (0.30 g L^−1^ day^−1^) was attained at 5 days of cultivation, while the maximum daily EPSs production (0.42 g L^−1^ day^−1^) was obtained at day 11 of the cultivation period. These findings demonstrate that the kinetics of the EPSs production and the kinetics of the cellular growth were dissociated, which is in concordance with many studies such as for *Porphyridium*, *Cyanothece* BH68K, or *Arthrospira* species [18,19].

Most EPSs are produced in the stationary phase and are considered secondary metabolites [1]. Still, EPSs production was observed during all growth phases but increased significantly during the stationary phase (Figure 1A), as is the case for *Anabaena flosaquae* A37, *Anabaena cylindrical*, or *Botryococcus braunii* [1,9]. However, few cyanobacteria strains, including *Nostoc*, are able to synthesize EPSs in more significant amounts during the exponential growth phase [20].

### 2.2. Chemical Composition Analysis and Monosaccharide’s Composition of G. gelatinosa EPSs

The freeze-dried *G. gelatinosa* EPSs were analyzed for their protein, carbohydrates, and lipid total contents by colorimetric analyses (Table 1). The chemical composition revealed that *G. gelatinosa* EPSs are principally composed of carbohydrates fraction. The total carbohydrates content was 70% of dry weight. The relatively high ratio of ester sulfated groups (10%) proposes that EPSs are sulfated polysaccharides in nature. The molar ratio hydrogen–carbon less than 2 and the presence of sulfur (0.5%) in the analyzed EPSs support this proposal. The present results agree with that found in the literature that revealed that several cyanobacterial EPSs are sulfated polysaccharides [1,9,17]. The presence of sulfate groups is also a feature of EPSs produced by eukaryotes and archaea but is rarely seen in bacterial EPSs [21].

**Figure 2:**
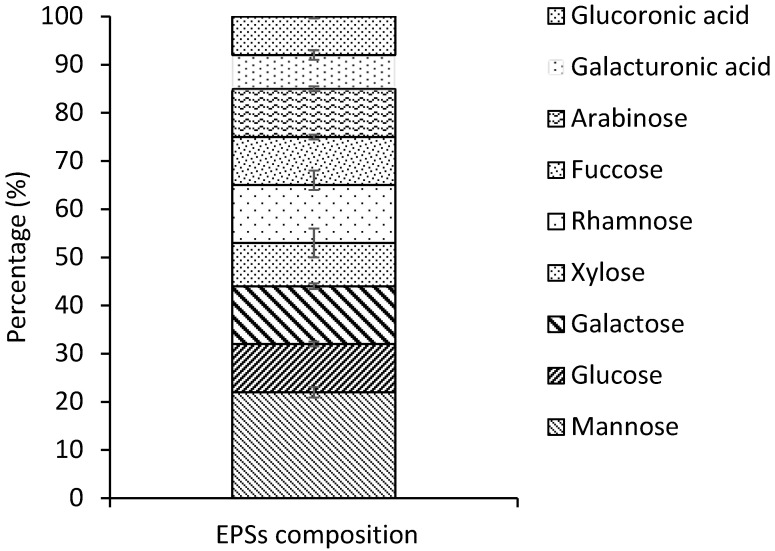
Monosaccharide composition of *G. gelatinosa* EPSs (%).

Furthermore, GC-FID analysis showed that *G. gelatinosa* EPSs contained at least nine different types of monosaccharides (Figure 2). The finding result illustrated a high proportion of hexoses (44 ± 3%; mainly mannose 22 ± 2%) and the presence of pentose’s (xylose; 9 ± 1% and arabinose; 10 ± 1%) usually absent in other EPSs of prokaryotic origin. This finding agrees with that observed for several strains counting *Nostoc* sp. and *Oscillatoria formosa* [14]. Other notable characteristics of the *G. gelatinosa* EPSs were the presence of two uronic acids (galacturonic; 7 ± 1% and glucuronic; 8 ± 0.1%), providing a high affinity for positively charged molecules (*e.g.*, metal cations) and the occurrence of deoxyhexoses (rhamnose; 12 ± 0.5% and fucose 10 ± 1%), enhancing the hydrophobicity of *G. gelatinosa* EPSs. These proportions are comparable to those found by Micheletti et al. [22] in the cyanobacteria *Gloeothece* sp. PCC 6909 EPSs with an amount of 13 and 11% for uronic acids and deoxyhexoses, respectively. Uronic acids were rarely found in the EPSs produced by other microbial groups [21].

The heterogeneous composition of the EPSs produced by *G. gelatinosa* agrees with the complexity found for cyanobacterial polymers [1]. The monosaccharide composition of most cyanobacterial EPSs can enclose up to 12 different residues [1]. However, EPSs with only four monosaccharides (ribose, xylose, glucose, and mannose) were also detected in Oscillatoria, Nostoc, and Cyanothece EPSs [1,21].

### 2.3. ^1^H NMR and FTIR Spectra of G. gelatinosa EPSs

**Figure 3:**
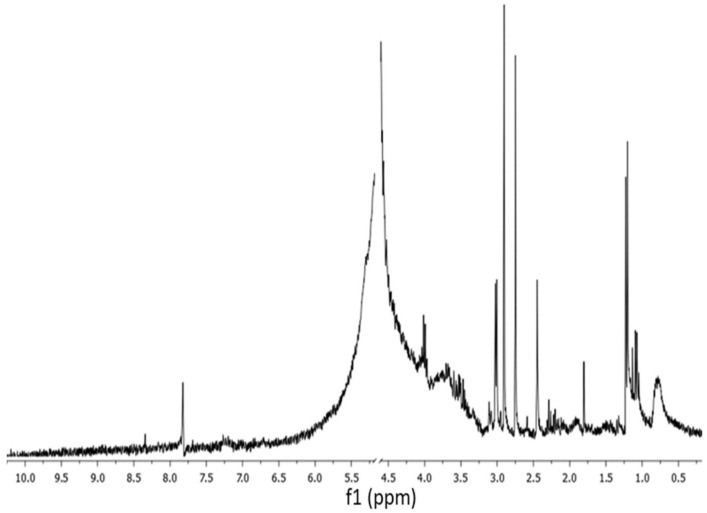
^1^H NMR spectra of *G. gelatinosa* EPSs. The peak at 5 ppm corresponding to the solvent was minimized.

*G. gelatinosa* EPSs structural analysis was also performed by ^1^H NMR and FTIR spectroscopy. The ^1^H NMR reveals the EPSs characteristic chemical shifts (Figure 3). The region of 3.2–4.5 ppm showed a crowded signal area due to many sugar residues, which is typical for polysaccharides. A broad signal corresponding to the H-6 of the rhamnose residues was also detected in the region of 1.2–1.4 ppm [23]. The signals detected at 2.2 and 2.7 ppm correspond to the acetyl amine group of hexoses or pentoses carbohydrates moiety [24]. A signal detected at 2.4 ppm could be assigned to uronic acids. The N–H group is also observed at 3.1 ppm. A signal located at 7.9 ppm was assigned to the o-acetyl ester groups [25].

**Figure 4:**
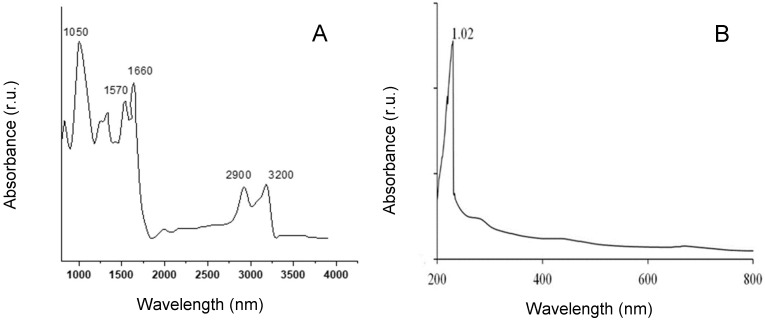
FTIR (**A**) and UV (**B**) spectra of *G. gelatinosa* EPSs.

The FTIR spectra of the EPSs produced by *G. gelatinosa* revealed several characteristic absorption bands (Figure 4A). A strong band was observed in the region of 3200 and 2900 cm^−1^, which could be attributed to the stretching vibration of O-H and C-H groups, respectively, typical of hydroxyl and alkyl functionality of carbohydrates [25]. Additionally, confirmed by UV spectra of the EPSs (Figure 4B) showed only a single peak at 210 nm, which is a common characteristic form of the electron transition of carbohydrates [25]. An important band was detected at 1050 cm^−1^ corresponding to the stretching vibration of ester sulfate groups [26]. Moreover, the presence of proteins was detected by the bands at 1660–1570 cm^−1^ attributed to N-H stretching [26]. The band at the region 1400 cm^−1^ corresponds to the C=O stretching vibration, which is a characteristic of a carboxyl group [27]. Overall, these findings strongly suggest that *G. gelatinosa* EPSs are heteropolysaccharides containing proteins and sulfated groups. However, additional separation steps to purify *G. gelatinosa* EPSs are required to obtain a full structure of the EPSs.

### 2.4. Molecular Weight and Zeta-Potential of G. gelatinosa EPSs

**Figure 5:**
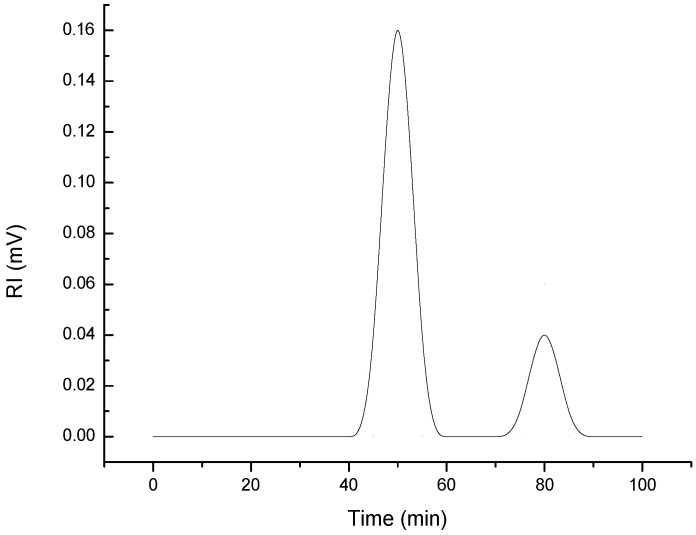
HPSEC chromatograms of *G. gelatinosa* EPSs.

The EPSs eluted from the HPSEC columns showed, among elution times, two distinctive peaks at 50 and 80 min (Figure 5), indicating that the *G. gelatinosa* EPSs have at least two polymer moieties, as generally found in the case of bacterial EPSs [28]. The first moiety (80% of the total area) eluted at 50 min has a molecular weight (Mw) of 598.3 ± 7.2 kDa. The second eluted at 80 min has a lower Mw evaluated at 67.2 ± 4.4 kDa. According to Alasonati and Slaveykova [28], the lower molar-mass fraction is generally predominated by protein-like substances, whereas the higher molar-mass fraction is rich in exopolysaccharides and exoproteins.

The zeta potential, an index of intensity of electrostatic (charge) attraction between particles, showed that the *G. gelatinosa* EPSs were anionic. The zeta potential was evaluated to −40 ± 2 mV. The negative charge may be due to the presence of anionic groups (e.g., COO–, C–O–, SO_4_^2−^) and to the high levels of glucuronic and galacturonic acid detected by the monosaccharide’s analyses. Consequently, *G. gelatinosa* EPSs could be quite reactive with other chemical species. The obtained results agree with several studies that showed that EPSs have a net negative which acts as an ion exchange matrix of cations [29].

### 2.5. Atomic Force Microscopy Analysis of G. gelatinosa EPSs

**Figure 6:**
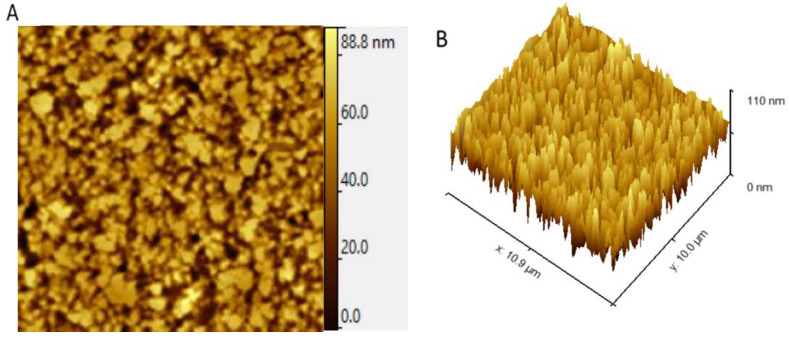
AFM 2D (**A**) and 3D (**B**) images of the *G. gelatinosa* EPSs.

AFM is a powerful tool to show the microstructure and morphological features of EPSs and thus help in understanding their physical properties. AFM microscopes are used in many different fields, including polymer science, semiconductor devices, thin films and coatings, energy storage, energy generation materials, biomolecules, cells and tissues, and many others [30]. 2D topographic AFM images (Figure 6A) revealed that the *G. gelatinosa* EPSs are composed of blocks of non-uniform size, which were different from previous observations of other bacterial EPSs with the globose-like [30] or fiber-like [31] structure. AFM 3D images (Figure 6B) revealed a compact structural feature with multimodal protrusions at a maximum height of 88 nm. The average roughness was evaluated at 0.28 nm. These characteristics account for strong inter/intra-molecular hydrogen bonds, and thus, *G. gelatinosa* EPSs may be suitable for application in various foods and water-holding capacity due to their compact structure [29].

### 2.6. Thermal Properties of G. gelatinosa EPSs

**Figure 7:**
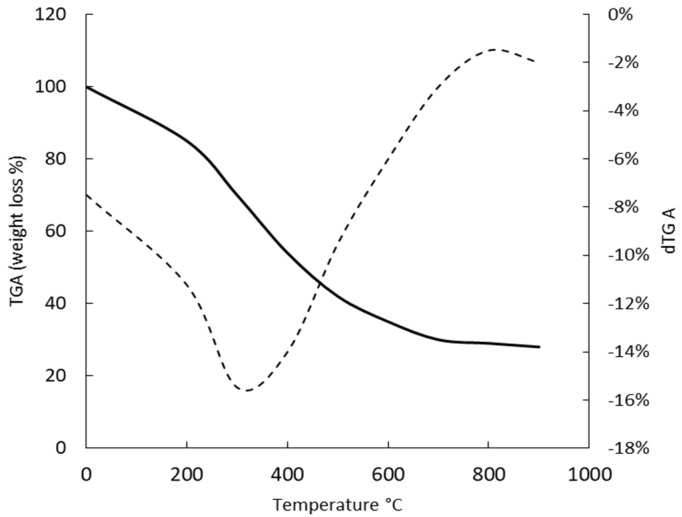
Thermogravimetric (TGA) curve (full line) and TGA derivative curve (dot lines) of the *G. gelatinosa* EPSs. The analysis was performed at a heating rate of 10 °C min^−1^ under a nitrogen atmosphere.

The decomposition pattern and the thermal stability of *G. gelatinosa* EPSs were studied by TGA (Figure 7). The freeze-dried EPSs revealed a minor weight loss, near 10%, when the temperature increases from 20 to 100 °C, corresponding to the water loss. A second degradation stage was observed at 100–600 °C with 50% weight loss near 400 °C and 70% around 600 °C. By analogy with natural gums, this degradation stage was attributed to the structural decomposition and pyrolysis of the polysaccharide moiety [32]. The final degradation stage was observed over the 650 °C, while even at temperatures greater than 700–800 °C, more than 23% remained solid residues. According to Chowdhury et al. [33], this stage was mainly due to the rupture of C–O and C–C bonds leading to CO and CO_2_ evaporation. The presence of uronic acids and sulfate groups prevents the total disaggregation of EPSs and explains the remaining solid residue [32].

Compared to other cyanobacteria (*Cyanothece* sp., *Oscillatoria* sp., *Nostoc* sp., *Nostoc carneum*) EPSs, where 50% of thermal degradation was observed at 275–300 °C [23,24], the *G. gelatinosa* EPSs seem of relatively good stability against temperature.

### 2.7. WHC and WSI Analysis

The WHC and WSI of *G. gelatinosa* EPSs were at 70.29 ± 2.36% and 97.43 ± 1.24%, respectively. To our knowledge, WHC and WSI of cyanobacterial EPSs, including *Gloeocapsa*, have not been reported. The WHC and WSI of EPSs produced by bacterial strains were respectively at 14.2–20.5% and 117–134% [34]. Compared to these values, *G. gelatinosa* EPSs show higher water holding capacity, which implies that the EPSs have a high potential to hold a mass of water mainly through hydrogen bonding. These properties are highly considered in the food industry, particularly as fat adsorber and flavor retention [34,35].

### 2.8. Metal Sorption Activity of G. gelatinosa EPSs

**Figure 8:**
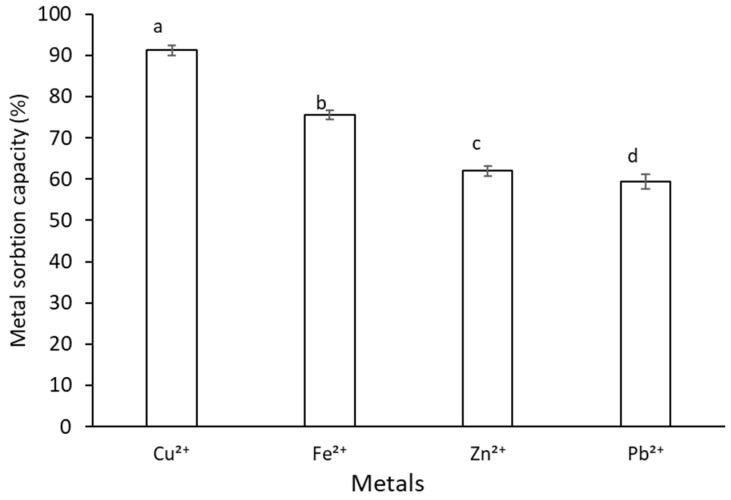
Metal sorption capacities of *G. gelatinosa* EPSs. Different letters mean statistically significant differences at *p* < 0.05. Each value is the mean of triplicate measurements. Water was used as a negative control.

*G. gelatinosa* EPSs bound significant amounts of bivalent heavy metal ions (Figure 8). EPSs have a high affinity toward Cu^2+^ and Fe^2+^ with metal sorption activities of 91.20 ± 1.25% and 75.51 ± 0.71%, respectively. Zn^2+^ and Pb^2+^ sorption efficiency was significantly lower, with metal sorption capacities of 62.03 ± 1.18% and 59.40 ± 1.02%, respectively. *G. gelatinosa* EPSs show a metal sorption activity similar to that recorded by several cyanobacteria and bacteria strains [35,36].

Indeed, at the same tested concentration, EPSs extracted from the thermophilic cyanobacteria *Leptolyngbya* sp. showed a high affinity toward Cu^2+^, Fe^2+^, Zn^2+,^ and Pb^2+^ with a metal adsorption capacities value of 93.25, 90.57, 87.33, and 84.29%, respectively.

The metal sorption activity of *G. gelatinosa* EPSs might be related to its anionic nature and/or to the binding metal ion’s ability of the O-H and C=O functional groups [35,36]. Thus, EPSs could be used as a flocculant to remove heavy metals from industrial wastewaters.

### 2.9. Viscosity of G. gelatinosa EPSs

**Figure 9:**
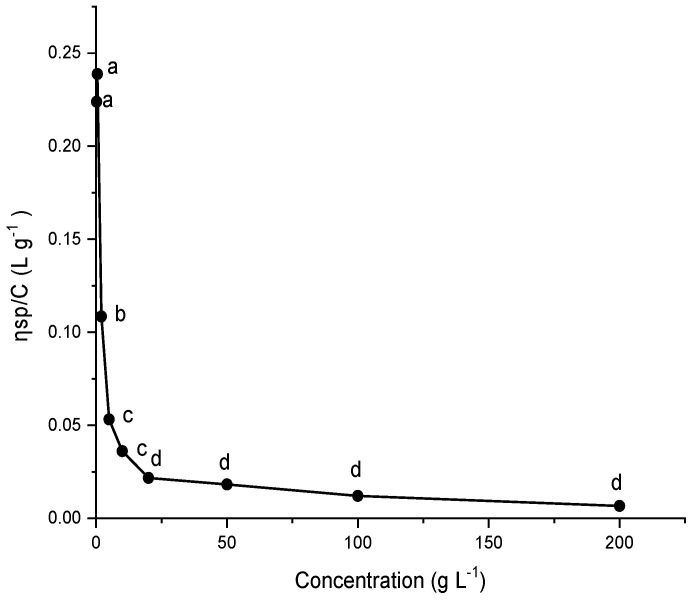
Plot of reduced viscosity of EPSs solutions for concentrations at 25 °C. Different letters mean statistically significant differences at *p* < 0.05. Each value is the mean of triplicate measurements. Water was used as a negative control.

Figure 9 shows the plots of reduced viscosity (ηsp/C) against the concentration of EPSs aqueous solutions. It is observed that the curve of reduced viscosity has an abnormal viscosity behavior showing a decrease in the viscosity when the EPSs concentration increases. This trend was observed for other charged polymers, including bacterial exopolysaccharides and polyvinyl alcohol [37]. It was attributed to the adsorption of polymeric solutes on the viscometer capillary walls reducing its effective radius and/or to the intermolecular electrostatic forces upswing the residual viscosity with decreasing the polymer concentration [36,37].

### 2.10. Antioxidant Capacity of G. gelatinosa EPSs

The antioxidant capacity is typically determined by several techniques involving direct or indirect measurements of the rate/extent of formation/decay of free radicals [38]. The antioxidant activity of *G. gelatinosa* EPSs was evaluated by four different indirect assays, namely inhibition of *β*-carotene bleaching, DPPH-radical-scavenging activities, iron-reducing activity, and iron-chelating capacity.

**Figure 10:**
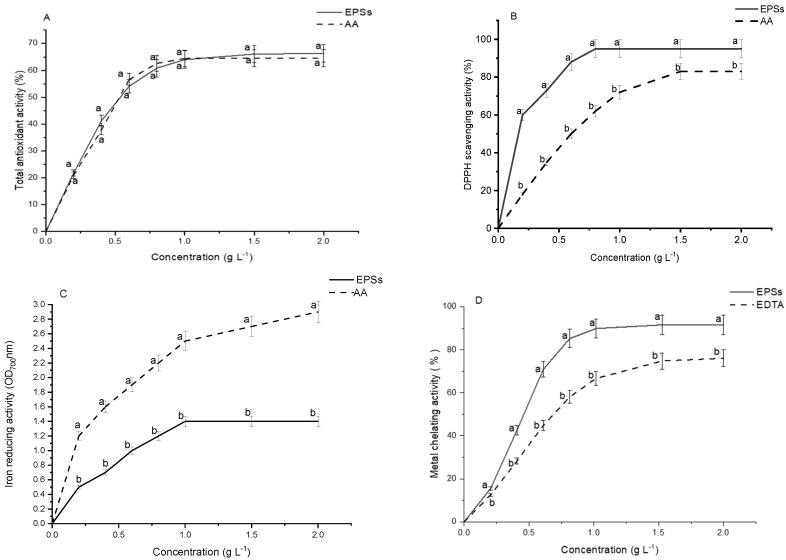
Antioxidant properties of *G. gelatinosa* EPS s aqueous solution at different concentrations: (**A**) total antioxidant activity; (**B**) DPPH-scavenging activity; (**C**) iron-reducing activity; (**D**) metal-chelating activity. AA and EDTA were used as positive controls. Different letters mean statistically significant differences at *p* < 0.05. Each value is the mean of triplicate measurements.

Total EPSs antioxidant activity was evaluated by the *β*-carotene bleaching effect induced in vitro by free radicals produced under the thermal oxidation of linoleic acid. As shown in Figure 10A, EPSs and AA exhibited a similar total antioxidant activity pattern, increasing significantly with the concentration increase. The IC_50_ value for both EPSs and AA was 0.5 g L^−1^.

The DPPH assays were used to test the ability of *G. gelatinosa* EPSs to donate H• and thus neutralize the reactive DPPH radicals. The present findings (Figure 10B) showed that the EPSs had a perceptible DPPH scavenging activity significantly higher than AA. G. EPSs scavenging activity varied from 60% at the concentration of 0.2 g L^−1^ and reached near 95% at 2 g L^−1^. The IC_50_ of *G. gelatinosa* EPSs on DPPH was 0.2 g L^−1^, three-fold lower than AA (0.6 g L^−1^). The weak dissociation energy of the polysaccharide O-H bond gives EPSs a high potential to donate H• implied in the stabilization of free radicals [38]. The hydrogen-donating ability was reckoned as the dominant antioxidant property of EPSs obtained from bacterial strains [38].

The reducing activity of EPSs was evaluated by measuring its ability to reduce ferric ion (Fe^3+^) to its ferrous form (Fe^2+^). Data (Figure 10C) showed that *G. gelatinosa* EPSs exhibited significantly lower electron-donating ability (OD_700_ nm = 1.0 at 2 g L^−1^) than AA (OD_700_ nm = 2.9 at 2 g L^−1^). Although the structural richness of cyanobacterial EPSs with various electron-donating functional groups (-OH, -SH, -COOH), the reducing activity could be expressed in the function of the species [6]. It seems that the difference in the configuration of the spatial polymer affects the degree of mobility of electrons and interferes with the charge density effects [5].

Nevertheless, the EPSs presented a higher metal chelating activity with an IC_50_ = 0.4 g L^−1^ than EDTA, used as a reference (IC_50_ = 0.6 g L^−1^). In both cases, the metal chelating activity was dose-dependent (Figure 10D). The maximum chelating activity was 91%, reached with 2 g L^−1^ of *G. gelatinosa* EPSs and only 75% for EDTA, under the same experimental conditions. Bacterial EPSs are well known for their binding and chelating capacity of several metal ions [22]. The high iron-chelating activity was also identified for several cyanobacterial EPSs [5,7], leading to the decrease in oxidation state and the suppression of the metal oxidant effects. Such findings demonstrated that EPSs constitute an environment that protects the cell against free radicals mainly produced at high temperatures. The in vitro results of antioxidant activity demonstrated that *G. gelatinosa* shows great potential to be developed as a natural antioxidant or functional food additive.

## 3. Materials and Methods

### 3.1. Organism and Culture Conditions

The current study involved a thermophilic cyanobacterium identified by phylogenetic analysis as *Gloeocapsa gelatinosa* (Culture Collection of National Institute of Marine Sciences, Tunisia). The strain was isolated from microbial mats anchored to submerged stones in hot water (60 °C) from a spring (Ain Echfa) located in the northern part of Tunisia (36°49′ N, 10°34′ E). Mats collected were treated by filtration, centrifugation, and dilution techniques according to standard microbiological protocols. The obtained axenic strain was identified morphologically as *Gloeocapsa gelatinosa* (*G. gelatinosa*) [39]. G. *gelatinosa* was grown in batch culture in 12 L capacity cylindrical bioreactors under optimal growth conditions, as suggested by Zili et al. [39]: BG_11_ medium; 40 ± 1 °C; 85 mole m^−2^ s^−1^ photons flux and 16:8 h light-dark cycles. The medium was sterilized by autoclaving at 120 °C for 20 min before inoculation. The strain was inoculated under sterile conditions with an initial cell density of 0.08 g L^−1^. The increase in the culture volume up to 12 L was carried out gradually by weekly subcultures in sterilized Erlenmeyer flasks. The purity of the experimental cultures was checked before and after inoculation by careful microscopic examination using phase contrast illumination (Carl Zeiss Axioskop 40, Oberkochen, Germany).

The biomass and EPSs daily productivity (Pt, g L^−1^ day^−1^) at any culture time (t) was calculated from Equation (1) where C_0_ and C_t_ as the dry mass density (g L^−1^) at start and time t (day), respectively.

(1) $$\mathrm{Pt}=\frac{C_{t\;}{-\;C}_{0\;}}{t}$$

### 3.2. Extraction and Purification of Extracellular Polymeric Substances

The *G. gelatinosa* EPSs were extracted from the stationary growth phase culture (day 12) according to Mezhoud et al. [12], where cultures were stirred gently for 30 min and then filtrated using a filter paper to detach the cells from the culture medium containing the released EPSs. The total recovered supernatant was concentrated using a tangential ultra-filtration cell (Vivaflow 50, Merck, Darmstadt, Germany) in Millipore membranes with 8 kDa pore size and then rinsed with deionized water until constant conductivity (0.08 m s^−1^) to eliminate low molecular weight substances and the media minerals. Finally, the filtrate was freeze-dried and weighed for EPSs content determination and stored at −20 °C for later characterization. Lyophilized EPSs were dissolved in double-distilled water to obtain aqueous EPSs solutions. Preliminary experiments showed that concentrations of over 300 g L^−1^ of *G. gelatinosa* freeze-dried EPSs can be fully dissolved in double-distilled water at 20 °C and with a pH of about 10.

### 3.3. Biochemical Composition and Monosaccharide Profile

The biochemical composition of the freeze-dried EPSs was determined using colorimetric and gravimetric methods. Total carbohydrates content was determined by the phenol sulfuric acid method according to DuBois et al. [40] using d-glucose (Sigma, Darmstadt, Germany, 50-99-7) as standard. Protein’s content was determined according to Lowry et al. [41] using bovine serum albumin (Sigma, Darmstadt, Germany, 10711454001) as standard. The ester sulfate groups were calorimetrically calculated using potassium sulfate as standard [42]. The total lipid content of EPSs was determined gravimetrically using the method of Folch et al. [43]. The EPSs elemental analysis (C, N, H, S, and P) was performed using a Flash Elemental Analyzer 1112 (ThermoQuest, Milan, Italy).

For the determination of monosaccharide profile [44], 5 mg of EPSs were hydrolyzed in 2 M Trifluoroacetic acid for 2 h at 100 °C and the released monosaccharides type GC 5890AGC 5890A (Hewlett Packard, Palo Alto, CA, USA) at 240 °C, and detection was performed via FID. The separation column RTX2330 (Restek, Bad Homburg, Germany) was used with a 30 m length and 25 μm diameter. Nitrogen and air-hydrogen mixture was used as the carrier gas and fuel, respectively. The extracts were subjected to derivatization before GC–MS analysis as described by Streeter and Strimbu [45], with the following modifications: 310 μL of pyridine and 250 μL of STOXX solution were used to resuspend dried samples, and 400 μL of HMDS and 40 μL of TFA were used to derivatize extracts. The separated monosaccharides were quantified using external calibration with an equimolar mixture of nine monosaccharide’s standards (analytical standard, Sigma Aldrich): hexoses (mannose, glucose, and galactose), pentoses (xylose and arabinose), deoxyhexoses (rhamnose and fucose), and acidic hexoses (galacturonic and glucuronic acids).

### 3.4. Proton Nuclear Magnetic Resonance Spectroscopy Analysis

The EPSs proton nuclear magnetic resonance spectroscopy (^1^H-NMR) was recorded in 25 µL deuterated water (D_2_O) on Bruker ASX400-WB spectrometer equipped with a double resonance (^1^H/X) Broad Band Inverse z-gradient probe head. Chemical shifts were expressed in ppm downfield from the signal of the methyl group of internal acetones (^1^H 2.225 ppm at 27 °C). The samples were analyzed in 5 mm susceptibility matched tubes (Shigemi, Japan). Integration of spectra and data analyses were performed with MESTRENOVA (version 2016).

### 3.5. Fourier Transform Infrared Spectroscopy and UV-Visible Spectroscopy Analyse

The Fourier transform infrared spectroscopy analysis (FTIR) spectra of the EPSs were performed in KBr using a Perkin–Elmer spectrum GX FTIR system (Perkin-Elmer, Maryland, USA). The spectra were recorded from 900 to 4000 cm^−1^, and 64 scans were averaged at a resolution of 4 cm^−1^. The UV-visible spectrum of the aqueous solution of EPSs (10 g L^−1^) was recorded at between 200 and 800 nm on a spectrophotometer (Beckman Coulter DU 640B, Derwood, MD, USA).

### 3.6. Determination of Molecular Weight

The molecular weight (Mw) of *G. gelatinosa* EPSs was determined according to the previously reported method [46]. Briefly, 2 mg of EPSs were dissolved in 2 mL of distilled water and heated at 75 °C for 15 min prior to analysis using the high-performance size-exclusion chromatography with a refractive index (RI) detection system (HPSEC-UVMALLS-RI system, Tokyo, Japan). Shodex OH-pak SB-805 column following an OH-Pak SB-G guard column (8 mm × 300 mm, Tokyo, Japan) was used at 25 °C. The column was eluted with phosphate buffer (50 mmol L^−1^) at a flow rate of 0.8 mL min^−1^. Standard dextran (0.3 g L^−1^; Sigma, Darmstadt, Germany, 31390) of molecular weights 5, 13.5, 22.6, 50 and 150, 300 and 600 kDa were used to build a calibration curve. The injection volume was 20 µL.

### 3.7. Zeta Potential

The zeta potential of *G. gelatinosa* EPSs aqueous solution at a concentration of 5 mg mL^−1^ was recorded using a sizer Nano ZSP (Malvern, New Jersey, USA). The measurements were performed in triplicate at 25 ± 1 °C. Each sample was determined with three independent measurements, containing 100 runs for each measurement.

### 3.8. Atomic Force Microscopy Analysis

The *G. gelatinosa* EPSs were examined under atomic force microscopy, AFM (NTEGRA Spectra, NT -MDT, Zelenograd, Russia) in tapping mode. The lyophilized EPS sample (0.1 mg mL^−1^) was fixed onto a cover mica slice surface and dried at room temperature.

### 3.9. Thermal Gravimetric Analysis

The thermal gravimetric analysis (TGA) of *G. gelatinosa* EPSs was evaluated on a Pyris 1 TGA (PerkinElmer, Texas, USA) according to the standard DIN ISO 11358. EPSs sample (20 mg) was heated, in the range of 20–900 °C, under a nitrogen atmosphere with a flow rate of 10 mL min^−1^ and a heating rate of 10 °C min^−1^. Data are presented as weight loss (%) as a function of temperature.

### 3.10. Water-Holding Capacity and Water Solubility Indexes

The water-holding capacity (WHC) and the water solubility index (WSI) of *G. gelatinosa* EPSs were measured according to Wang et al. [47] with minor modifications. The lyophilized *G. gelatinosa* EPSs sample (50 mg) was suspended in 10 mL of distilled water and kept in a water bath for 30 min at 50 °C. The obtained solution was then centrifuged at 11,000× *g* for 30 min. The EPSs pellets were filtered on pre-weighed filter paper to remove moisture completely. Then, the filter paper was weighed and recorded for WHC determination. The supernatant obtained from the centrifugation process was poured into a petri dish and dried at 110 °C for 8 h, and the dry solid weight was used to determine the WSI. WHC and WSI were evaluated using Equations (2) and (3):

(2) $$\mathrm{WHC}\%=\frac{\mathrm{Total}\;\mathrm{sample}\;\mathrm{weight}\;\mathrm{after}\;\mathrm{water}\;\mathrm{absorption}\;}{\mathrm{total}\;\mathrm{dry}\;\mathrm{weight}\;\mathrm{sample}}\;\times \;100\;$$

(3) $$\mathrm{WSI}\%=\frac{\mathrm{weight}\;\mathrm{of}\;\mathrm{dry}\;\mathrm{sample}\;\mathrm{solid}\;\mathrm{in}\;\mathrm{the}\;\mathrm{supernatant}}{\mathrm{weight}\;\mathrm{of}\;\mathrm{the}\;\mathrm{dry}\;\mathrm{sample}}\;\times \;100\;$$

### 3.11. Metal Sorption Activity

The metal sorption activity of *G. gelatinosa* EPSs was measured based on Abed et al. [7]. Volumes of 50 mL of Cu^2+^, Fe^2+^, Zn^2+^ or Pb^2+^ metal solution (10 mg L^−1^) were mixed separately with 2 mL (10 mg L^−1^) EPSs solutions. Mixtures were then incubated in the dark at 25 °C for 5 h and separated by centrifugation at 10000× g for 10 min at 4 °C. An atomic absorption spectrometer (iCE 3500 Thermo Scientific, Waltham, MA, USA) was used to determine the residual metal concentrations.

Each metal sorption activity was evaluated as the proportion (%) of residual to initial metal concentrations.

### 3.12. Viscosity Measurements

Absolute viscosities (η) of *G. gelatinosa* EPSs solutions were measured at 25 °C using a Brookfield viscometer (DV2TRVTJO, Waltham, MA, USA). In each experiment, from 0.05 to 200 mg of crude EPSs were dissolved in 10 mL of distilled water.

The reduced viscosity was evaluated by Equation (4), where (ηr) is the relative viscosity evaluated using the formula ηr = $\frac{\eta }{\eta 0}$ · η represents the absolute viscosity of the EPSs, and ηo is the absolute viscosity of distilled water. C is the EPSs concentration.

(4) $$\frac{\eta \mathrm{sp}}{C}(\mathrm{mL}\;g^{-1})=\frac{\eta r^{-1}}{C}$$

### 3.13. Screening of the Antioxidant Capacity

#### 3.13.1. Total Antioxidant Activity

Total antioxidant activity was studied using the *β*-carotene bleaching test based on Dapkevicius et al. [48]. The emulsion of *β*-carotene/linolenic acid was freshly prepared by suspending 0.5 mg of *β*-carotene and 25 µL of linolenic acid and 200 µL of tween 20 (Sigma, Darmstadt, Germany, 85114) in 1 mL chloroform. Then, all the solvent was evaporated under vacuum in a rotary evaporator at 55 °C. After that, 100 mL of oxygenated water was added to the mixture and mixed. Subsequently, 2.5 mL of the prepared solution was added to 500 µL of *G. gelatinosa* EPS aqueous solution (concentrations ranged from 0 to 2 g L^−1^). The control sample tubes were prepared by adding only 500 µL of water. The tubes were maintained in a water bath for 2 h at 55 °C, and the UV/Vis absorbance was measured in triplicate at 470 nm. L-ascorbic acid (AA) was evaluated with the same procedure and used as a standard. Tests were carried out in triplicate, and the antioxidant activity was evaluated in terms of β-carotene bleaching using Equation (5), where OD_0_ and OD_t_ are the absorbances of the test sample measured before and after incubation, respectively, and OD_0’_ and OD_t’_ are the absorbencies of the control sample measured before and after incubation, respectively. The IC_50_, defined as the EPSs samples’ concentration that prevents 50% of β-carotene bleaching, was determined.

(5) $$\mathrm{Total}\;\mathrm{antioxidant}\;\mathrm{activity}\;(\%)=[1-\frac{{\mathrm{OD}}_{0}{-\mathrm{OD}}_{t}}{{\mathrm{OD}}_{0}{-\mathrm{OD}}_{t}}]\;\times \;100\;$$

#### 3.13.2. α,α-diphenyl-β-picrylhydrazyl Radical Scavenging Activity

The α,αdiphenyl-β-picrylhydrazyl radical-scavenging activity (DPPH) was evaluated according to the method described by Bersuder et al. [49] with slight modifications.

*G. gelatinosa* EPSs aqueous solution in different concentrations range (from 0 to 2 g L^−1^) were mixed with 500 µL of a methanolic solution containing DPPH (0.02%). Next, the mixture was incubated for 1 h in the dark at room temperature. A control containing DPPH without EPSs was also prepared. The absorbance was measured at 517 nm and normalized to a blank sample consisting of DPPH solution. The scavenging activity of DPPH was determined by Equation (6):

(6) $$\mathrm{DPPH}\;\mathrm{radical}\;\mathrm{scavenging}\;\mathrm{activity}\;(\%)=\frac{A517\;\mathrm{control}-A517\;\mathrm{sample}}{A517\;\mathrm{control}}\;\times \;100\;$$

The same procedure was made with AA as standard. The IC_50_, defined as the concentration of EPSs samples that scavenged 50% of DPPH radical, was determined.

#### 3.13.3. Iron-Reducing Activity

The ability of *G. gelatinosa* EPSs to reduce iron (Fe^3+^) was determined by the method reported by Adjimani and Asare [50]. Briefly, 0.5 mL of *G. gelatinosa* EPSs aqueous solution (concentrations ranged from 0 to 2 g L^−1^) or AA (used as standard) was mixed with 1.25 mL of potassium phosphate buffer (0.2 M, pH 7) and 1.25 mL of 1% potassium ferricyanide solution. The mixtures were incubated for 30 min in a 50 °C water bath. After cooled, the resulting solution was added with 0.5 mL of 10% trichloroacetic acid and centrifuged at 3000 g for 10 min. The supernatant (1.25 mL) from each sample mixture was then mixed with 0.5 mL of 0.1% ferric chloride. The blank sample was prepared following the same experimental procedure where the distilled water was used instead of the ferric chloride solution. After allowing the reaction to proceed for 10 min, the UV/Vis absorbance was measured at 700 nm. Higher absorbance values indicated greater reducing power. Three replicates were used for each sample.

#### 3.13.4. Metal-Chelating Activity

The metal-chelating activity of the different samples was determined according to the method reported by Wettasinghe and Shahidi [51]. Briefly, 100 µL of *G. gelatinosa* EPSs aqueous solution (concentrations ranged from 0 to 2 g L^−1^) was added to 50 μL of ferrous chloride (2 mM) and 450 μL of methanol. After 5 min of incubation at room temperature, the reaction was started by adding 200 µL of ferrozine solution (5 mM). The mixture was stirred and left to react for 10 min to allow the iron complexation. Control samples were prepared following the same experimental procedure replacing EPSs with water and EDTA (used as a standard). The UV/Vis absorbance of the solution was measured at 562 nm. The metal-chelating activity was expressed as a percentage using Equation (7), where OD_c_, OD_b_, and OD_s_ represent the absorbance of the control, the blank, and the sample, respectively. The test was performed in triplicate, and the IC_50_ was evaluated.

(7) $$\mathrm{Metal}\;\mathrm{chelating}\;\mathrm{activity}\;\left(\%\right)\;=\frac{{\mathrm{OD}}_{C}{\;+\;\mathrm{OD}}_{b}{\;-\;\mathrm{OD}}_{s}}{{\;\mathrm{OD}}_{c}\;}\;\times \;100\;$$

### 3.14. Statistical Analysis

The results were expressed as means ± standard deviation (SD) of three replicates. Statistical analysis of the data was carried out using the software SPSS Statistics 20. Differences between treatments were assessed with Student’s *t*-test, and the *p* values < 0.05 were considered statistically significant.

## 4. Conclusions

The *G. gelatinosa* EPSs can play an important role in various biotechnological and industrial applications. In this study, *G. gelatinosa* EPSs were produced in optimal growth conditions in a 12 L cylindrical reactor. The results show that G. *gelatinosa* is a competent EPSs producer, releasing most EPSs at the stationary growth phase. The EPSs produced by this strain were composed mainly of carbohydrates, having nine different monosaccharides, including two uronic acids, and revealed the presence of ester sulfate groups and proteins. The EPSs were composed of two polymers fractions with different molecular weights, and they are remarkably thermostable and with anionic nature. AFM images showed a non-uniform topography with a pointed compact structural feature. They have high water-holding and metal sorption activities. The potent antioxidant activity of *G. gelatinosa* EPSs was marked by a great DPPH-radical-scavenging and high metal-chelating activities. This work highlights the potential of the thermophilic *G. gelatinosa* as a novel bioactive natural resource to sustainable biotechnological processes. It represents a viable alternative to promote the use of the warm waters of thermal springs by their recovery in ecological and safe cyanobacteria biomass production systems. Nevertheless, further research is needed to ensure that *Gloeocapsa gelatinosa* is a non-producing cyanotoxins species and that EPSs exhibit no toxicity and to assess the economic feasibility before mass production.

## Figures and Tables

**Table 1:** *G. gelatinosa* EPSs chemical composition: carbohydrates, protein, lipid, ester sulfate groups, and elemental analysis (% in EPSs dry weight).

Lipids	Carbohydrates	Proteins	Ester Sulfate	C→N	H→S→P
0.8 ± 0.1	70.2 ± 0.1	12.2 ± 0.02	10.8 ± 0.06	30.5→16.5	21.1→0.5→0.06

## Data Availability

All data generated or analyzed during this study are included in the article.
